# Rare Form of Erdheim-Chester Disease Presenting with Isolated Central Skeletal Lesions Treated with a Combination of Alfa-Interferon and Zoledronic Acid

**DOI:** 10.1155/2015/876752

**Published:** 2015-04-08

**Authors:** E. N. Bulycheva, V. V. Baykov, M. I. Zaraĭskiĭ, G. N. Salogub

**Affiliations:** ^1^First Pavlov State Medical University of St. Petersburg, L'va Tolstogo Street, No. 6/8, Saint Petersburg 197022, Russia; ^2^University Hospital Carl Gustav Carus, Fetscherstrasse 74, 01307 Dresden, Germany

## Abstract

Erdheim-Chester disease (ECD) represents a clonal non-Langerhans histiocytosis, which manifests under an extensive variety of clinical symptoms. This creates a challenge for the physician, who is required to recognize and diagnose the disease in the early stages. Despite this considerable challenge, in the last decade there has been a dramatic increase in ECD diagnoses, in most part due to an increasing awareness of this rare disorder. Involvement of the axial skeleton is exclusively uncommon with no official recommendations for the treatment of the bone lesions. Here, we present a case report of a young male patient with isolated lesions of the spine, ribs, and pelvis, who was successfully treated with a combination therapy of alfa-interferon and zoledronic acid.

## 1. Introduction

Erdheim-Chester disease (ECD) is a rare non-Langerhans histiocytosis of unknown etiology, characterized by focal or systemic tissue infiltration of foamy histiocytes, which have a particular tropism towards connective tissue. These cells typically share the immunophenotype FXIIIa+, CD68+, CD163+, CD1a−, and S100−. The clinical picture ranges from mild asymptomatic infiltration to severe cases, which are sometimes life-threatening. The disease is typically entrenched in the long bones, but it may also affect the central nervous system, retroperitoneal organs, the orbit, lungs, and other organs. The spinal vertebrae are usually spared in ECD with only two cases reported [1]. To date, the consensus guidelines for the diagnostics and treatment of ECD have been collected and established [2], although the recommendations regarding the therapeutic approach to bone lesions have not been thoroughly discussed. It has been postulated that the prognosis of the patient is determined by the extent and the distribution of the extraskeletal manifestations of the disease; simultaneously, data recording the clinical progression of the patients with isolated bone lesions are lacking.

## 2. Case Presentation

**Figure 1:**
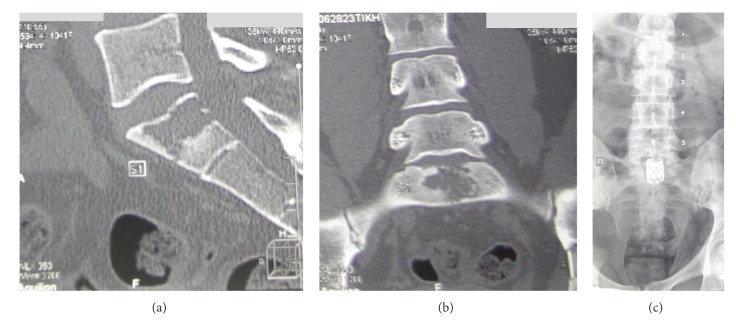
Sagittal (a) and frontal (b) CT showing osteolytic bone lesions in S1, sized 2.9∗2.1∗2.2 cm. (c) Frontal X-rays of lumbosacral region of spine, showing spinal fusion with titanic block.

A 28-year-old man presented in the clinic with pain in his lower back and ribs. The patient had a history of Hodgkin lymphoma at the age of 9 years, with complete clinical and hematological remission after combined chemo- and radiotherapy. It was recorded that the patient's ailments, at the time of presentation, had commenced at the age of 23 years, and 3 years later the osteolytic bone lesions in S1 and L5 vertebrae were found on MRI and CT scans (Figures 1(a) and 1(b)). No data of tuberculous osteitis was obtained. The patient underwent a surgical resection of the S1 vertebral body and spinal fusion with titanic block (Figure 1(c)). Six months later, due to the progression in thoracic vertebrae, the tunnel resection and radiothermoablation of Th4 and Th7 were performed.

**Figure 2:**
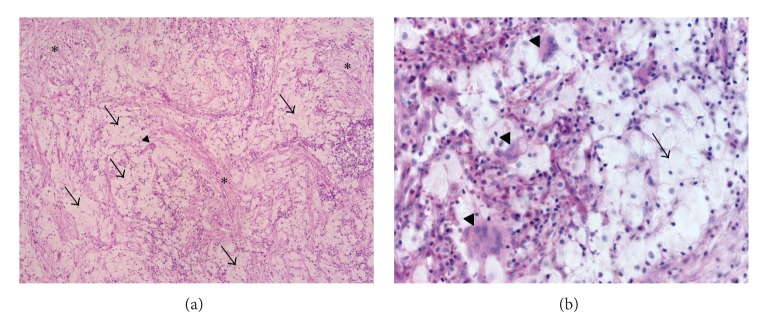
(a) Hematoxylin-eosin staining, ×100. (b) High-power view of hematoxylin-eosin staining, ×400. Arrows: groups of xanthoma cells, arrowhead: giant multinuclear cell, stars: foci of fibrosis.

**Figure 3:**
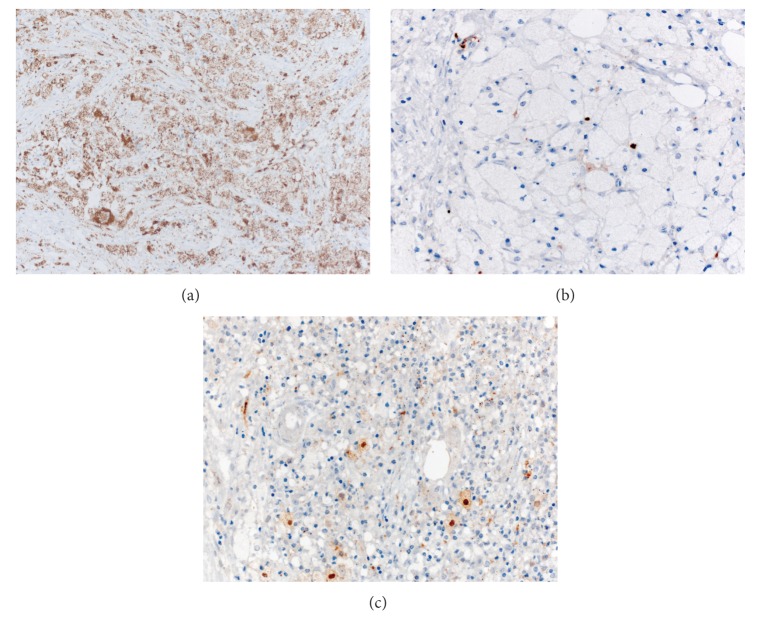
Immunohistochemistry. (a) Staining for CD68, ×200. Prominent expression of CD68 in xanthoma and giant multinuclear cells. (b) Staining for S-100, ×400. Less than 5% of the xanthoma cells express S-100. (c) Staining for FXIIIa, ×400. 10–15% xanthoma cells have nuclear-cytoplasmic expression of FXIIIa.

Postoperative material was prepared for histological investigation and subsequent analysis revealed the foci of fibrosis, within which there were groups of xanthoma cells and multinuclear giant cells (Figures 2(a) and 2(b)); the Reed-Sternberg cells were not detected. These cells stained positively for CD68 (Figure 3(a)), but not CD1a. Less than 5% of the cells were S100 positive (Figure 3(b)), and 10–15% were positive for factor XIIIa (Figure 3(c)). Such an immunophenotype suggested that ECD was a plausible explanation for the reported symptoms, and the relapse of Hodgkin disease could be excluded as atypical cells with CD30 and CD15 expression were not detected. At the PET-scan, multiple foci of increased FDG uptake were identified in C5, Th7–Th11, L3, L5 vertebrae, 3rd, 7th, and 8th left ribs, the right pubic bone, and both iliac bones, with a maximal size of 2.4∗3.9 cm in L3 and maximal SUV of 12.5 in Th7. Additional examination revealed autoimmune thyroiditis with hypothyreosis. Therefore, we initiated the therapy with L-thyroxine. In order to exclude the typical organ involvement in ECD, we performed an MRI scan of the central nervous system, which revealed no abnormalities. An ultrasound of the abdomen, echocardiography, and thoracic CT were also performed, which collectively demonstrated no signs of aortic involvement, retroperitoneal fibrosis, or any other pathological findings. Radiological investigation of the long bones revealed neither osteolytic nor osteopetrotic lesions; ophthalmological status was normal. There were no signs of systemic inflammation and CRP and BSG levels remained normal throughout the entire observation period. The BRAF V600E mutation was detected in bone marrow and peripheral blood by MASA (mutant allele-specific amplification) and is described in more than 50% of ECD cases [3, 4]. The detailed description of MASA technique is provided in Supplement 1 in Supplementary Material available online at http://dx.doi.org/10.1155/2015/876752.

Taking into account the foci of bone destruction with signs of progression, bone pain, and verified diagnosis of ECD, a therapy plan was commenced with alfa-interferon 3 million units 3 times per week, combined with zoledronic acid 4 mg once monthly. This therapy is now well tolerated with only a moderate flu-like syndrome observed, which is controlled by paracetamol and mild neutropenia, which does not require therapy with colony-stimulating factors. At follow-up, 2 years after the start of the therapy the patient is pain-free and his overall quality of life has now much increased. The PET-scan was repeated and revealed that smaller foci (maximal size – 1.5∗2.3 cm in L3) of increased FDG uptake persisted only in Th7–Th9, L1, and L3, with maximum SUV of 1.6. This affirmed a marked improvement in the course of this patient's disease.

## 3. Discussion

This case report presents ECD diagnosed at an early stage with unusual spine and pelvic involvement, without extraskeletal manifestations. It is of interest that the patient had a lymphoproliferative disease in his childhood, but the potential relationship between these two diseases remains to be elucidated. To date, there is only one published case report where ECD occurs after acute lymphoblastic leukemia in childhood [5]. An early diagnosis is important in order to start disease-modulating therapy, which may improve prognosis and survival of patients, and is of significant importance to active clinical studies recruiting for this disorder. At the time of diagnosis, specific therapy utilizing a BRAF inhibitor (vemurafenib), which may be the best therapeutic option in the presence of the BRAF mutation, was not available in the Russian Federation. Moreover, experience with BRAF inhibition is lacking in the treatment of ECD worldwide, although the current results of this treatment option in a small patient cohort appear promising with an objective of sustained clinical improvement and good tolerability [6]. Other therapeutic options for this patient could be to prevent the biologic activity of the proinflammatory cytokines, with a recombinant, nonglycosylated form of the human IL-1 receptor antagonist (anakinra), or infliximab, an antagonist of TNF-alpha; however, taking into account the expected life-long treatment and corresponding financial expenses, this treatment was considered to be unacceptable. Furthermore, the expected side effects of these drugs together with the lack of systemic inflammation throughout the monitored course of the disease strengthened our decision against this treatment. It is known that alfa-interferon enhances the terminal differentiation of histiocytes and dendritic cells [7] and many clinical cases have shown its efficacy [8–11]. Therefore, the administration of this drug in our patient with ECD seemed to be a logical, promising treatment. Due to the lack of other systemic involvements, we also combined interferon with the aminobisphosphonate (zoledronic acid), based on good clinical outcomes in previously described patients receiving this therapy [12–14]. This clinical case shows a very good response and tolerability of this treatment combination, implying further investigation of its safety and duration of response.

## Supplementary Material

Description of the mutant allele-specific PCR amplification method (MASA-PCR) to detect the mutant BRAF gene.
