# Supplementary material for: Rare Form of Erdheim-Chester Disease Presenting with Isolated Central Skeletal Lesions Treated with a Combination of Alfa-Interferon and Zoledronic Acid
Source: Case Rep Hematol. 2015 Apr 8;2015:876752. doi: 10.1155/2015/876752 (PMC4408628; doi:10.1155/2015/876752)
Supplement: Supplementary file 1 — Description of the mutant allele-specific PCR amplification method (MASA-PCR) to detect the mutant BRAF gene. [file 876752.f1.pdf]

Supplement 1. Detection of the mutant BRAF gene

DNA was isolated from 250 μl blood plasma by column method using a K-Sorb set (Moscow, Russia) according to the manufacturer's instructions. As a basic technique for the detection of the mutant BRAF gene we have chosen MASA-PCR (mutant allele-specific PCR amplification). This technique is more sensitive than direct sequencing and SSCP (single-strand conformation polymorphism) (1). MASA-PCR was conducted as described previously (2), combined with SYBR green-based real-time PCR. Briefly, two different forward primers 5`-TAGGTGATTTTGGTCTAGCTACAGT-3` (as a positive control to amplify wild-type as well as mutant BRAF) and 5`-GGTGATTTTGGTCTAGCTACAAA-3` (to amplify mutant BRAF gene only) were used. The sequence of the reverse primer was 5`-GGCCAAAATTTAATCAGTGGA-3`. Each reaction mixture contained 10.0 μl of 2× SYBR green quantitative PCR Mix (Moscow, Russia), 0.5 μM of forward and reverse primers, and 2.0 μl of DNA and was made up to a total volume of 20.0 μl with DNase-free water. Cycling was performed on a iCycler Thermal Cycler (BioRad, USA) with the following two-step cycling conditions: an initial enzyme activation step at 95°C for 2 min, followed by 40 cycles of denaturation at 95°C for 10 s and primer annealing and elongation at 60°C for 30 s, with the fluorescence signal being read on green at 60°C following extension.

References

1. Sapio MR, Posca D, Troncone G, et al. (2006) Detection of BRAF mutation in thyroid papillary carcinomas by mutant allele-specific PCR amplification (MASA). Eur J Endocrinol. 154 (2): 341-8.
2. Collins BJ, Schneider AB, Prinz RA, Xu X. (2006) Low frequency of BRAF mutations in adult patients with papillary thyroid cancers following childhood radiation exposure. Thyroid 16(1): 61-6.
